# Impact of Metformin on Systemic Metabolism and Survival of Patients With Advanced Pancreatic Neuroendocrine Tumors

**DOI:** 10.3389/fonc.2019.00902

**Published:** 2019-09-20

**Authors:** Claudio Vernieri, Sara Pusceddu, Filippo de Braud

**Affiliations:** ^1^Fondazione IRCCS Istituto Nazionale dei Tumori, Milan, Italy; ^2^IFOM, The FIRC Institute of Molecular Oncology, Milan, Italy; ^3^Oncology and Hematology-Oncology Department, University of Milan, Milan, Italy

**Keywords:** pancreatic neuroendocrine tumors, metformin, metabolism, glucose, lipid metabolism, diabetes mellitus

## Introduction

**Figure 1:**
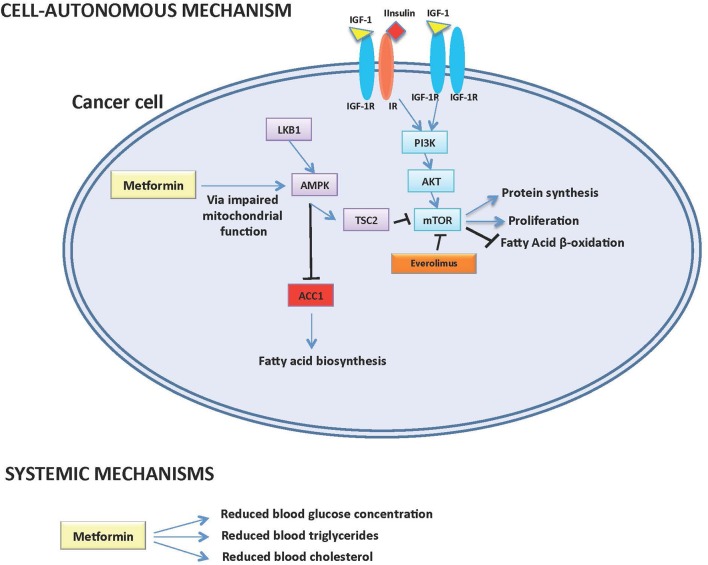
Potential cell-autonomous and systemic antitumor mechanisms of action of Metformin in pNETs.

Pancreatic neuroendocrine tumors (pNETs) represent a subgroup of neuroendocrine malignancies with specific biological and clinical characteristics, whose incidence has increased in the last four decades. The growth and proliferation of pNET cells is especially dependent on the IGF-1/IGF1-receptor/PI3K/AKT/mTOR signaling pathway, while the activation of somatostatin receptor axis exerts antiproliferative effects [Figure 1; (1, 2)].

In recent years, the following clinical achievements dramatically expanded the therapeutic armamentarium against advanced pNETs: (a) somatostatin analogs (SAs) significantly prolonged patient progression free survival (PFS) when compared to the placebo in patients with advanced disease (3); (b) the mTOR inhibitor everolimus and the multi-tyrosine kinase inhibitor sunitinib demonstrated anticancer activity and prolonged median PFS in pNET patients pre-treated with SAs (4, 5); (c) peptide receptor radiotherapy (PRRT) showed impressive anticancer activity coupled with excellent tolerability profiles in patients with pre-treated advanced pNETs. Finally, cytotoxic chemotherapy or liver-directed treatments remain a valid option for patients with high-grade pNETs, as well as for neoplasms progressing on biological agents. Despite these improvements, most advanced pNETs remain almost invariably incurable, and many patients finally die of their disease. Indeed, while 5-year survival is 65–94% in patients with limited-stage disease, it is reduced to 44–76% in the presence of lymph node metastases, and to 27% in the case of distant metastases (6, 7). In the advanced disease setting, factors associated with poorer survival include the presence of liver and peritoneal metastases, not having undergone resection the primary (pancreatic) tumor, high-grade (G3) disease. Therefore, new treatment options are needed for patients with advanced pNETs, in particular those with poor prognostic factors.

In recent years, preclinical and retrospective clinical data have shown that the antidiabetic compound metformin may have antitumor activity against different tumor types including pNETs. Potential mechanisms of metformin anticancer effects include: (1) modifications of systemic metabolism, including a reduction of blood glucose and insulin, which sustain cancer cell growth by fueling cell metabolism; (2) direct, cell-autonomous anticancer effects, which are mediated by the inhibition of mitochondrial metabolism and ATP production, with the consequent impairment of intracellular energetic status and inhibition of mTOR, protein and fatty acid biosynthesis. However, the potential anticancer role of metformin in patients with advanced pNETs remains to be fully elucidated.

Here we review and discuss preclinical and clinical studies supporting a potential role of metformin in the treatment of pNETs. We also discuss how ongoing trials could elucidate a potential role of metformin in combination with established anti-pNET therapies.

## Methods

We searched in PubMed database using the following string: “(biguanides OR metformin) AND (pancreatic neuroendocrine tumors OR pNETs OR neuroendocrine tumors.)” Our search strategy produced a total number of 82 articles. Among them, we selected for this review article those original preclinical and clinical papers that investigated the metformin in preclinical pNET models or in patients with advanced pNETs.

### Evidence of Metformin Activity Against pNETs

#### Preclinical Evidence

Metformin has demonstrated anti-pNET activity in preclinical studies (8–10). For instance, metformin impaired cell migration capacity and reduced the survival of BON-1 (serotonin-secreting) and QGP-1 (non-hormone-secreting) pNET cell lines (8). In BON-1 but not in QGP-1 cells, metformin strongly inhibited the transcription of insulin receptor gene (*INSR*), and also reduced levels of phosphorylated ERK and AKT (8). In another study, metformin inhibited the mTORC1/S6K/S6 pathway when used in the 1–10 mM concentration range, and reduced cell viability without inducing apoptosis (9). Due to the central role of the INSR-IGFR1/PI3K/AKT/mTOR pathway in pNET cell growth and proliferation, the ability of metformin to inhibit this axis via AMPK activation may be responsible for its cell-autonomous anticancer effects, as well as for a potentially synergistic antitumor activity between mTORC1 inhibitors and metformin.

Nevertheless, the following issues strongly limit the clinical translatability of *in vitro* studies published so far: (1) metformin concentrations used in cell growth media are in the range of mM (usually 1–20 mM), i.e., by far higher than those that can be reached in patient blood with commonly-used and safe metformin dosages 4–15 μM (11). This limitation highlights the importance of repeating crucial experiments with more physiological metformin concentrations; (2) the contribution of metformin-induced systemic metabolism modifications on its anticancer activity cannot be assessed in *in vitro* studies. Since metformin may inhibit cancer growth by modifying systemic metabolism, and in particular by lowering the blood concentration of glucose, insulin, and lipids, this limitation is especially important in the perspective of their clinical translation.

#### Clinical Data

The first indication of a potential impact of metformin on the outcome of patients with advanced pNET patients came from a small retrospective study that we conducted in 31 patients. In this study, we found that metformin use in diabetic patients was associated with significantly longer PFS when compared to diabetic patients not receiving metformin, or to non-diabetic patients (Table 1)(12).

**Table 1:** Published and ongoing studies of metformin in pNET patients.

**References**	**Study design**	**N. patients**	**Status**	**Study findings**
Pusceddu et al. (12)	Retrospective	31	Completed	Diabetic pNET patients taking metformin have significantly longer PFS when compared with non-diabetics or diabetics pNET patients treated with other antidiabetic therapies
Pusceddu et al. (13)	Retrospective	445	Completed	Diabetic pNET patients taking metformin have significantly longer PFS (44.2 months) when compared with non-diabetics (15.1 months) or diabetics pNET patients treated with other antidiabetic therapies (20.8 months). The impact of metformin on patient PFS was independent of other clinically relevant variables
Vernieri et al. (14)	Retrospective	58	Completed	Hypertriglyceridemia and hypercholesterolemia are associated with significantly worse PFS in advanced pNET patients treated with everolimus
Pusceddu et al. (NCT02294006) (14)	Prospective	43	Ongoing	N.A.

*PFS: progression-free survival; pNET: pancreatic neuroendocrine tumor*.

Aiming to expand these preliminary data, we conducted a large retrospective, multicentric study involving 24 Italian centers and 445 patients (13). In this study, we found that diabetic patients treated with metformin had remarkably longer PFS (44.2 months) when compared to other diabetics (20.8 months) or to non-diabetic patients (15.1 months) (Table 1). Importantly, the positive impact of metformin was observed regardless of the concomitant anticancer treatment (SA or everolimus plus SA), and was independent from other known prognostic variables, including the presence of liver metastases or having undergone previous surgery of the primary tumor. In our study, patient glycemic status was not independently associated with PFS, thus suggesting that plasma glucose levels do not affect treatment efficacy (13). We also provided indirect arguments supporting the conclusion that blood insulin concentration is unlikely to have an effect on patient outcomes. Therefore, we finally hypothesized that the anticancer role of metformin in advanced pNETs is more likely to be mediated by cell-autonomous antitumor effects.

On the other hand, we recently published results of a study indicating that modifications of lipid metabolism could be implicated in metformin anticancer properties. Indeed, in a 58 patients with advanced pNETs treated with everolimus, we found that the precocious (within 3 months from treatment initiation) onset of hypertriglyceridemia, or increased cholesterol levels during the whole treatment course, are associated with significantly lower PFS independently from metformin use (Table 1) (14). We also found that high intratumor levels of Acetyl-CoA Carboxylase 1 (ACC1) enzyme, the limiting-step enzyme in the fatty acid *de novo* biosynthesis pathway, correlate with lower everolimus efficacy [Figure 1; (14)]. Since metformin not only affects systemic glucose metabolism, but it is also capable of lowering plasma triglycerides (15) and/or of causing inhibition of ACC1 in AMPK-mediated manner in cancer cells (16), the observed association of metformin use and significantly longer patient PFS could be mediated by metformin effects on systemic/tumor lipid metabolism (Figure 1). Prospective studies are needed to test this hypothesis, as well as to distinguish between an indirect (i.e., mediated by modifications of systemic metabolism) and a direct, cell-autonomous anticancer effect of metformin in pNETs.

#### Ongoing Studies

While retrospective analyses clearly indicate that metformin use in diabetic patients with advanced pNETs is associated with better clinical outcomes, no prospective studies have investigated metformin activity/efficacy in combination with standard antitumor treatments so far. Moreover, it is currently unclear if also pNET patients who are not diabetics ma benefit from metformin treatment.

T2DM is characterized by the concomitancy of hyperglycemia, insulin resistance, and hyperinsulinemia (17). Furthermore, T2DM is frequently associated with metabolic syndrome, which is defined by the presence of glucose intolerance, hypertriglyceridemia, low HDL cholesterol levels, obesity, and high blood pressure (18). Therefore, the presence of both glucose and lipid metabolism dysregulation is common in T2DM patients (17). Metformin is effective in reducing hyperglycemia and insulin resistance occurring in T2DM patients, and also reduced blood triglyceride and cholesterol concentration in some studies (15).

At our Institution, the single-arm, open label MetNet1 trial (NCT02294006) is currently enrolling patients with advanced pNETs regardless of their diabetic status (19). Patients enrolled in this trial are prescribed upfront treatment with SAs plus everolimus plus metformin, up to a maximum daily dosage of 2,000 mg. The primary objective of the study is to evaluate the efficacy of the experimental treatment, as defined as median PFS. Other study objectives consist in testing the tolerability of the experimental treatment, as well as its effects on systemic metabolism. Of note, metformin does not significantly alter glucose and lipid metabolism in patients with normal baseline profiles. Therefore, if metformin anticancer effects in pNET patients are mainly mediated through modifications of systemic metabolism, diabetic pNETs patients, who more frequently have deregulated glucose and lipid metabolism, may benefit from metformin significantly more than non-diabetic ones. Conversely, if metformin mainly acts through a cell-autonomous anticancer effect, diabetic, and non-diabetic patients should benefit from metformin treatment in a similar way. Discarding between these two possibilities will be crucial to properly select pNET patients who are the best candidates to receive metformin in combination with standard anticancer treatments.

## Discussion

Based on the available preclinical and retrospective clinical evidence, metformin administration promises to provide clinical advantage when used in combination with established anticancer treatments, such as SAs and everolimus, in patients with advanced pNETs (12–14). Since plasma glucose levels have not been found to be associated with pNET patient prognosis, it is unlikely that the major effect of metformin is mediated by its ability to reduce patient glycemia. On the other hand, emerging data suggest that the effect of metformin could be mediated through its impact on systemic lipid metabolism, especially in patients treated with mTOR inhibitors, which increase triglyceride and cholesterol concentration in a significant proportion of patients [Figure 1; (14)]. However, these data need to be confirmed in larger retrospective and, in case, in prospective studies. Moreover, a direct, cell-autonomous anticancer effect of metformin against pNETs cannot be excluded, even though metformin concentrations that are active in *in vitro* studies can be hardly reached in patients' blood (Figure 1).

Crucial advantages of metformin consist in low drug costs and excellent tolerability at dosages that are commonly used for the treatment of T2DM. However, the tolerability of metformin in combination with standard anticancer treatments needs to be established yet. For instance, the metformin-everolimus combination could increase the risk of everolimus-induced diarrhea. The ongoing NCT02294006 trial will clarify if metformin is a safe and well-tolerated drug when combined with SAs plus everolimus (19). While the risks of specific pharmacological metformin-including combinations cannot be ignored, metformin could also prevent or reduce alterations of glucose and lipid metabolism that are often detected in patients with advanced pNETs, especially those treated with everolimus (4, 14).

While published clinical studies indicate a potentially relevant advantage from adding metformin to standard anti-pNET treatments, prospective studies are necessary before concluding that metformin might provide a true clinical benefit. Indeed, retrospective studies have important limitations that may lead to incorrect conclusions. For instance, metformin use had been associated with longer survival in patients with advanced pancreatic exocrine adenocarcinomas in retrospective studies (20, 21); however, three recent randomized trials showed no benefit from adding metformin to first- or second-line chemotherapy in this patient population (22–24). Different factors may account for discrepancies between retrospective and prospective studies, including the reporting bias, immortal time bias, and the fact that metformin is only taken by patients with T2DM in retrospective studies (13, 25). In the case of pNETs, the impact of the immortal time bias could be especially important: indeed, pNET patients receiving metformin for T2DM treatment could be selected for being exposed to SAs and/or everolimus for longer periods, or for having undergone previous pancreatic surgery, i.e., all clinical characteristics associated with better patient prognosis independently from metformin use.

Another crucial issue in the debate around the use of metformin as an anticancer agent consists in clarifying its potential antitumor activity in patients who are not diabetics. Since in our retrospective study the diabetic status was not associated with patient PFS independently from other prognostic factors, it is reasonable to hypothesize that metformin could improve patient prognosis independently from its impact on glucose metabolism but, more reasonably, through its effects on other metabolic pathways or through cell-autonomous anticancer effects. In both cases, we would expect similar anticancer activity from metformin in patients with and without diabetes. Prospective studies including both diabetic and non-diabetic patients, as well as correlative analyses between kinetics of blood triglyceride/cholesterol concentration and treatment efficacy, will be crucial to clarify the role of metformin-induced metabolic modifications on its anticancer activity. On the other hand, preoperative, window-of-opportunity trials with single-agent metformin in patients candidate to surgery could represent the ideal context to explore potential metformin cell-autonomous antitumor properties, as well as to clarify if commonly used dosages of this compound are sufficient to reach therapeutic intratumor concentrations.

To date, the strongest rationale exists for combining metformin with everolimus, which could synergize at a molecular (i.e., by strengthening inhibition of the PI3K/AKT/mTOR pathway and inhibiting cancer cell anabolism) and systemic (i.e., by reducing blood glucose, triglyceride, and cholesterol concentration) levels. However, future preclinical and, in case, clinical studies should investigate metformin in combination with other therapies that are standard-of-care in pNET patients, such as SSAs and PPRT.

## Author Contributions

All authors (CV, FB, and SP) have contributed to conception or design of the paper, as well as to the writing of the manuscript and its critical revision.

### Conflict of Interest Statement

The authors declare that the research was conducted in the absence of any commercial or financial relationships that could be construed as a potential conflict of interest.
